# 

**Published:** 1880-08

**Authors:** 


					AUGUST, 1880.
THE
AMERICAN JOURNAL
op
DENTAL SCIENCE,
EDITED BY
F. J. S. GORGAS, M. D? D. D. S?
AND
JAMES B. HODGKIN, D. D. S.
VOL. 14.?THIRD SERIES.?No. 4.
BALTIMORE:
SNOW DEN & COWMAN.
LONDON:
TKUBNER & CO., GO PATERNOSTER ROW.
1 8 8 0.
PAYABLE IN ADVANCE.
?
a
p)
w
w
c/2;
I1
P-i'
Ox
o
<-0
t?a
>-
ss
tzj
d
tsg?

				

